# Transovarial transmission of *Borrelia* spp., *Rickettsia* spp. and *Anaplasma phagocytophilum* in *Ixodes ricinus* under field conditions extrapolated from DNA detection in questing larvae

**DOI:** 10.1186/s13071-020-04049-7

**Published:** 2020-04-07

**Authors:** Daniela Hauck, Daniela Jordan, Andrea Springer, Bettina Schunack, Stefan Pachnicke, Volker Fingerle, Christina Strube

**Affiliations:** 1 Institute for Parasitology, Centre for Infection Medicine, University of Veterinary Medicine Hannover, Buenteweg 17, 30559 Hanover, Germany; 2 Bayer Animal Health GmbH, 51373 Leverkusen, Germany; 3 Bayer Vital GmbH, 51368 Leverkusen, Germany; 4 National Reference Centre for Borrelia, Veterinaerstraße 2, 85764 Oberschleissheim, Germany

**Keywords:** *Borrelia burgdorferi* (*sensu lato*), *Borrelia miyamotoi*, Rickettsiales, *Anaplasma phagocytophilum*, Ticks, Tick-borne diseases

## Abstract

**Background:**

*Ixodes ricinus* constitutes the main European vector tick for the Lyme borreliosis pathogen *Borrelia burgdorferi* (*sensu lato*), the relapsing fever borrelia *Borrelia miyamotoi*, as well as *Anaplasma phagocytophilum* and several *Rickettsia* species. Under laboratory conditions, a transovarial transmission to the next tick generation is described for *Rickettsia* spp. and *Borrelia* spp., especially regarding *B. miyamotoi*, whereas the efficiency of transovarial transfer under field conditions is largely unstudied.

**Methods:**

In order to better estimate the potential infection risk by tick larvae for humans and animals, 1500 *I. ricinus* larvae from 50 collected “nests” (larvae adhering to the flag in a clumped manner) were individually examined for *Borrelia*, *Rickettsia* and *A. phagocytophilum* DNA using quantitative real-time PCR (qPCR).

**Results:**

Thirty-nine of 50 nests each (78.0%, 95% CI: 64.0–88.5%) were positive for *Borrelia* spp. and *Rickettsia* spp. DNA, and in three nests (6.0%, 95% CI: 1.3–16.5%) *A.* *phagocytophilum* DNA was detected. Overall, DNA from at least one pathogen could be detected in 90.0% (45/50, 95% CI: 78.2–96.7%) of the nests. Of the 1500 larvae, 137 were positive for *Borrelia* spp. DNA (9.1%, 95% CI: 7.7–10.7%), 341 for *Rickettsia* spp. DNA (22.7%, 95% CI: 20.6–24.9%) and three for *A.* *phagocytophilum* DNA (0.2%, 95% CI: 0–0.6%). Quantity of *Borrelia* spp. and *Anaplasma* spp. DNA in positive larvae was low, with 2.7 × 10^0^*Borrelia 5S-23S* gene copies and 2.4 × 10^1^*A. phagocytophilum msp2/p44* gene copies detected on average, while *Rickettsia*-positive samples contained on average 5.4 × 10^2^*gltA* gene copies. Coinfections were found in 66.0% (33/50, 95% CI: 51.2–78.8%) of the nests and 8.6% (38/443, 95% CI: 6.1–11.6%) of positive larvae. In fact, larvae had a significantly higher probability of being infected with *Borrelia* spp. or *Rickettsia* spp. when both pathogens were present in the nest.

**Conclusions:**

This study provides evidence for transovarial transmission of *Rickettsia* spp. and *Borrelia* spp. in *I. ricinus* under field conditions, possibly facilitating pathogen persistence in the ecosystem and reducing the dependence on the presence of suitable reservoir hosts. Further studies are needed to prove transovarial transmission and to explain the surprisingly high proportion of nests containing *Rickettsia* and/or *Borrelia* DNA-positive larvae compared to infection rates in adult ticks commonly reported in other studies.
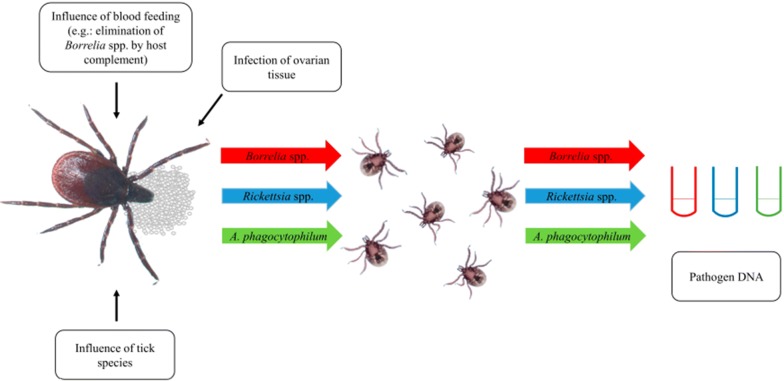

## Background

*Ixodes ricinus* is the most widespread tick species in Europe and acts as a vector for a range of bacterial and viral tick-borne pathogens with relevance for human and animal health. In Germany, different genospecies of the *B.* *burgdorferi* (*sensu lato*) complex, *B.* *miyamotoi*, *A.* *phagocytophilum* and *Rickettsia* spp. are among the most important bacterial pathogens transmitted by *I. ricinus*. These pathogens cause various diseases in humans and animals. *Borrelia* *burgdorferi* (*s.l.*) is the causative agent of Lyme borreliosis (LB), while *B.* *miyamotoi* causes febrile illness and has been associated with meningoencephalitis in immunocompromised patients in Europe [1, 2]. Furthermore, several *Rickettsia* spp. may cause spotted fever or lymphadenopathy in humans [3]. In *I. ricinus*, *R.* *helvetica*, *R.* *monacensis*, *R.* *massiliae* and *R.* *felis* have been detected, with *R.* *helvetica* being the most frequent [3–7]. Another member of the order Rickettsiales, *A. phagocytophilum*, may cause granulocytic anaplasmosis in humans, dogs, horses, goats, sheep and cattle [8–10].

Transmission of these pathogens between or in ticks may occur in various ways, including co-feeding, transstadial, sexual as well as transovarial transmission. Co-feeding transmission, whereby infection spreads from one tick to another feeding in close proximity on the host, seems to be very rare [11–13]. Transstadial transmission, i.e. the transmission of pathogens from one developmental stage to the next, occurs in *Borrelia* spp. as well as *Rickettsia* spp. and *A. phagocytophilum* [14–17]. In contrast, sexual transmission, i.e. transmission from a male to a female tick during copulation, has only been described for *Rickettsia* species as well as some relapsing-fever borreliae [18, 19]. During transovarial transmission, the offspring of an infected female is affected. An efficient transovarial transmission rate of up to 100% has been shown for *Rickettsia* spp. under laboratory conditions [17, 20], whereas efficiency is generally believed to be much lower for *Borrelia burgdorferi* (*s.l.*), which are mainly transmitted to larvae *via* a blood meal from an infected host or rarely by co-feeding [21–23]. *Borrelia miyamotoi*, on the other hand, can be transmitted transovarially from the female tick to more than 90.0% of its larvae [22, 24]. In contrast, a low to inefficient transovarial transmission rate has been described for *A. phagocytophilum* [25–27]. Transovarial transmission facilitates the persistence of pathogens in the ecosystem and thus reduces dependence on suitable reservoir hosts.

The main hosts of *I. ricinus* larvae are rodents [28], but humans may also serve as accidental hosts [29]. Many prevalence studies have examined pathogen prevalence in nymphs and adult ticks, but only a few have examined tick larvae. Therefore, the aim of this study was to determine *Borrelia* spp., *Rickettsia* spp. and *A. phagocytophilum* DNA in questing *I.* *ricinus* larvae to extrapolate potential transovarial transmission rates of these pathogens under natural conditions and assess the possible human or animal infection risk due to *I.* *ricinus* larvae.

## Methods

### Tick larvae collection and molecular species identification

**Fig. 1:**
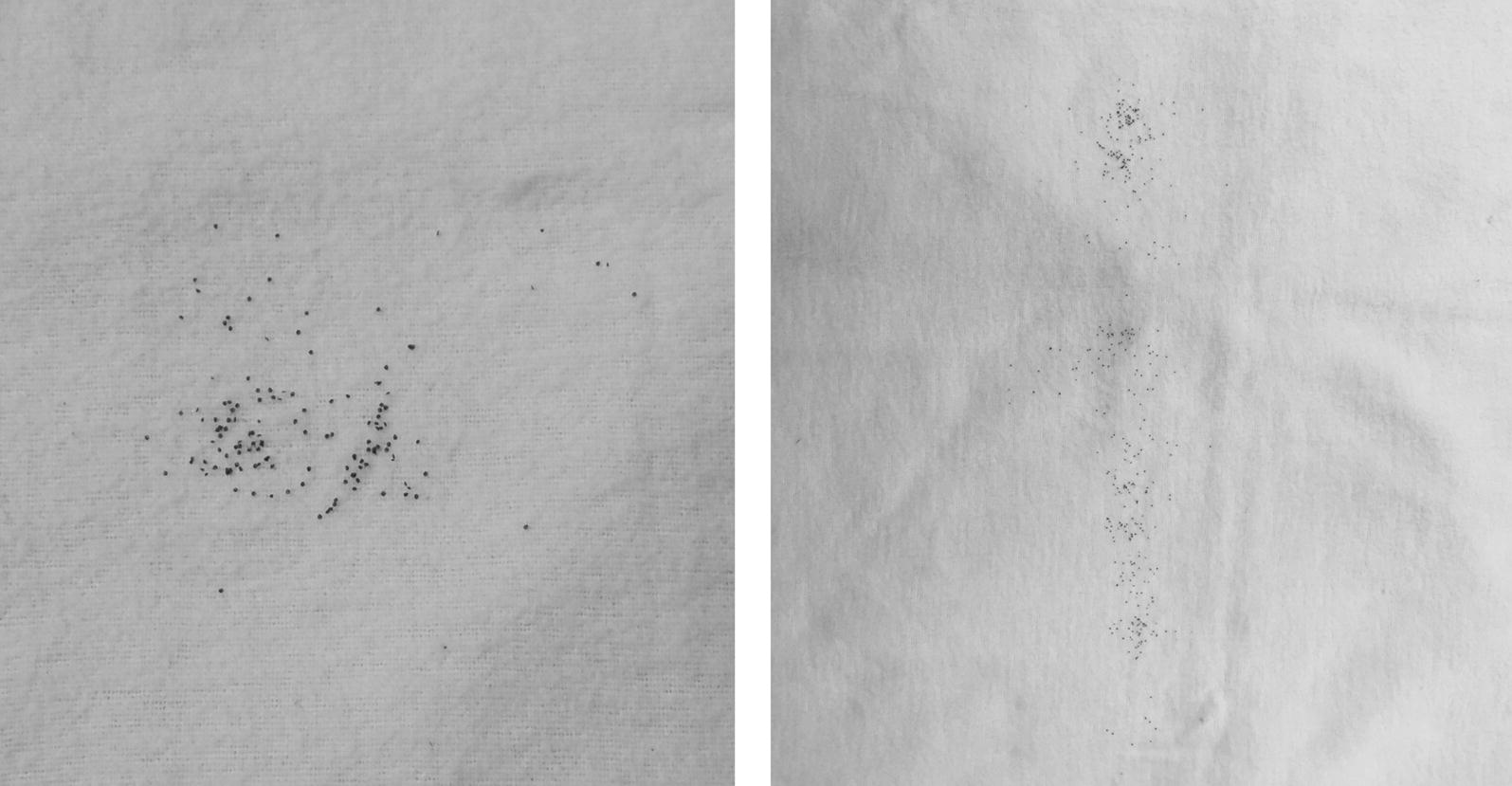
Representative examples for larvae adhering to the flag in a clumped manner, which were defined as an *Ixodes* spp. nest

Questing tick larvae were collected by the flagging method at different sampling sites in northern Germany during 2010–2018. The flag was dragged approximately one meter over the ground, and all larvae adhering to the flag in a clumped manner were defined as a “nest” originating from a single female (Fig. 1). After freezing the flags with the collected larvae overnight at − 20 °C, larvae were picked off the flag and stored individually at − 20 °C until genomic DNA isolation.

Tick species identification was performed on genomic DNA of two larvae per nest by amplification and sequencing of a part of the *16S* rRNA gene, using primers described by Mangold et al. [30]. The reaction mixture and thermoprofile were set up as described by Hauck et al. [31], except that the amount of DNA template was increased to 6 μl and the number of PCR cycles to 41. Amplicons were separated by electrophoresis on 1.5% agarose gels stained with GelRed® (Biotium Inc., Fremont, CA, USA) and visualized under UV light. Obtained PCR products were Sanger-sequenced at Microsynth Seqlab Laboratories (Göttingen, Germany) and aligned with *16S* rRNA gene sequences published by Estrada-Peña et al. [32] [GenBank: KM211785, KM211786, KM211787, KM211788 (*I. ricinus*); KM211789, KM211790 (*I. inopinatus*)] as well as other selected sequences of *I. ricinus*, *I. inopinatus* and *I. frontalis* available on GenBank [accession nos. GU074592, GU074605 (*I. ricinus*); KY569415, KY569416, KY569417, KY569418 (*I. inopinatus*), MF688050 (*I. frontalis*)] using Clone Manager 9 Professional Edition (Scientific & Educational Software, Denver, USA).

### Tick larvae testing for bacterial pathogen DNA

In total, 1500 larvae originating from 50 larvae nests were examined for *Borrelia* spp., *Rickettsia* spp. and *A. phagocytophilum*. Per nest, 10–40 larvae (average 30 larvae per nest, standard deviation, SD: 11.6) were examined, depending on nest size. In case of a small nest (≤ 40 larvae) all larvae were examined, whereas for larger nests 40 larvae were tested. Larvae were individually homogenized using 0.5 ml polysterene pistils (VWR, Darmstadt, Germany). Genomic DNA was extracted from individual tick larvae using the Nucleo Spin® 8 Blood Core Kit (Macherey-Nagel, Düren, Germany) according to the manufacturer’s specifications, with previously described amendments [5]. Until further use, isolated genomic DNA was stored at − 20 °C.

Testing for *Borrelia* spp. (*B. burgdorferi* (*s.l.*) and *B. miyamotoi*) and *Rickettsia* spp. by duplex quantitative real-time PCR (qPCR) was carried out as described previously [33, 34]. For *Borrelia* spp., the *5S*–*23S* rRNA intergenic spacer (IGS) region was targeted based on a primer-TaqMan™ minor groove binder (MGB) probe combination designed by Strube et al. [35]. For detection of *Rickettsia* spp., the citrate synthase (*gltA*) gene was amplified based on a primer-TaqMan™ probe combination by Stenos et al. [36]. The reaction set-up and thermal cycling were performed as described previously [5]. Regarding *A. phagocytophilum*, larvae nests from 2010 and 2011 were tested by targeting the *16S* rRNA gene using a primer-TaqMan™ probe combination by Sirigireddy and Ganta [37]. At the same time, successful DNA isolation was confirmed by duplex amplification of the *Ixodes* ITS2 region as previously described [5]. For larvae nests collected from 2015 to 2018, the *msp2/p44* gene with a primer-TaqMan™ probe combination by Courtney et al. [38] was targeted. Again, successful DNA isolation was confirmed by simultaneous amplification of the *Ixodes* ITS2 region. The duplex reaction set-up and thermal cycling were carried out according to Blazejak et al. [7].

### *Borrelia* (geno-)species and *Rickettsia* species identification

To determine the (geno-)species of *Borrelia*-positive tick larvae, the Reverse Line Blot (RLB) technique was performed for larvae nests from 2010/2011 by amplifying a fragment of the *B. burgdorferi* (*s.l.*) *5S-23S* rRNA IGS region using biotin-linked forward primer 5SCB and reverse primer 23SN2 as published by Tappe et al. [39]. For larvae collected during 2015–2018, the biotin-linked forward primer B5S was used instead of 5SCB, and a second, hydrolase-23S rRNA region specific biotin-linked forward primer was added for specific amplification of *B.* *miyamotoi* as described by Blazejak et al. [40]. Moreover, the RLB setup was modified to include the BisNE1 probe as described by Springer et al. [29].

For *Rickettsia* species identification, a subset of the *Rickettsia*-positive samples was subjected to real-time pyrosequencing of a sequence stretch of the rickettsial citrate synthase (*gltA*) gene as previously described [41].

### Statistical analyses

Statistical analyses were conducted in R v. 3.3.1 [42]. To assess factors influencing larval prevalence of *Borrelia* spp. and *Rickettsia* spp., generalized linear mixed effect models (GLMMs) with binomial error structure were constructed. *Anaplasma phagocytophilum* prevalence was not statistically analysed because of the low number of positive larvae. The fixed factors were “location/year” (Hanover 2010, 2015, 2017, 2018, Mellendorf 2018 and Hamburg 2011) and “coinfection of the nest” (yes/no). Due to the small number of nests originating from some locations, only nests from Hanover, Mellendorf and Hamburg were included (*n* = 1220 larvae from 43 nests). Furthermore, “nest ID” was included as a random effects term. Each model was compared to a null model including only the random effects term in a likelihood ratio test (R function ‘anova’, test = ‘chisq’).

Finally, the predictive variable “location/year” was subjected to *post-hoc* analysis, computing all pairwise differences between factor levels in a Tukey’s test based on the parameters of the fitted GLMM.

## Results

### Tick larvae collection and molecular species identification

Total nest size of the 50 collected nests varied between 10 and 1643 larvae. Most nests were collected in Hanover (20/50), Hamburg (12/50) and Mellendorf (11/50). Detailed numbers of larvae per nest including collection sites and dates are presented in Table 1. Molecular species identification revealed that all collected nests belonged to *I. ricinus*.

**Table 1:** Detection of pathogenic microorganisms in *I. ricinus* larvae nests

Nest	Location (Sampling site)	Sampling month	No. of examined larvae (total number of larvae)	*Borrelia* spp. *n*/*N* (%)^a^	*Rickettsia* spp. *n*/*N* (%)^a^	*A. phagocytophilum* *n*/*N* (%)^a^	Coinfection *Borrelia* spp. + *Rickettsia* spp. *n*/*N* (%)	Coinfection *Rickettsia* spp. + A. *phagocytophilum* *n*/*N* (%)
1	Hanover (Misburger Wald)	May 2010	20 (20)	3/20 (15.0)	1/20 (5.0)	0/20 (0)	1/20 (5.0)	0/20 (0)
2	Hanover (Bornumer Holz)	Jun 2010	28 (28)	2/28 (7.1)	10/28 (35.7)	0/28 (0)	0/28 (0)	0/28 (0)
3	Hanover (Seelhorster Wald)	Jun 2010	34 (34)	2/34 (5.9)	0/34 (0)	0/34 (0)	0/34 (0)	0/34 (0)
4	Hanover (Mecklenheide)	Jun 2010	12 (12)	0/12 (0)	0/12 (0)	0/12 (0)	0/12 (0)	0/12 (0)
5	Hanover (Misburger Wald)	Aug 2010	11 (11)	0/11 (0)	0/11 (0)	0/11 (0)	0/11 (0)	0/11 (0)
6	Hanover (Georgengarten)	Aug 2010	10 (10)	0/10 (0)	0/10 (0)	0/10 (0)	0/10 (0)	0/10 (0)
7	Hanover (Alte Heide)	Sep 2010	28 (28)	1/28 (3.6)	1/28 (3.6)	0/28 (0)	0/28 (0)	0/28 (0)
8	Hamburg (Schwarzberg)	May 2011	15 (15)	1/15 (6.7)	1/15 (6.7)	0/15 (0)	0/15 (0)	0/15 (0)
9	Hamburg (Oejendorfer Park)	May 2011	20 (20)	9/20 (45.0)	5/20 (25.0)	0/20 (0)	2/20 (10.0)	0/20 (0)
10	Hamburg (Raakmoor)	Jun 2011	18 (18)	5/18 (27.8)	1/18 (5.6)	0/18 (0)	0/18 (0)	0/18 (0)
11	Hamburg (Neugrabener Heide)	Jun 2011	16 (16)	0/16 (0)	0/16 (0)	0/16 (0)	0/16 (0)	0/16 (0)
12	Hamburg (Schwarzenberg)	Jun 2011	13 (13)	2/13 (15.4)	0/13 (0)	0/13 (0	0/13 (0)	0/13 (0)
13	Hamburg (Gosselers Park)	Jun 2011	20 (20)	7/20 (35.0)	1/20 (5.0)	0/20 (0)	1/20 (5.0)	0/20 (0)
14	Hamburg (Stadtpark Winterhude)	Jun 2011	19 (19)	1/19 (5.3)	15/19 (78.9)	0/19 (0)	1/19 (5.3)	0/19 (0)
15	Hamburg (Oejendorfer Park)	Jun 2011	19 (19)	1/19 (5.3)	6/19 (31.6)	0/19 (0)	0/19 (0)	0/19 (0)
16	Hamburg (Gosselers Park)	Jul 2011	16 (16)	1/16 (6.3)	1/16 (6.3)	0/16 (0)	1/16 (6.3)	0/16 (0)
17	Hamburg (Altonaer Volkspark)	Jul 2011	15 (15)	1/15 (6.7)	1/15 (6.7)	0/15 (0)	0/15 (0)	0/15 (0)
18	Hamburg (Alster)	Jul 2011	16 (16)	3/16 (18.8)	7/16 (43.8)	0/16 (0)	0/16 (0)	0/16 (0)
19	Hamburg (Stadtpark Winterhude)	Jul 2011	20 (20)	2/20 (10.0)	13/20 (65.0)	0/20 (0)	1/20 (5.0)	0/20 (0)
20	Hanover (Bothfeld)	May 2015	12 (12)	2/12 (16.7)	2/12 (16.7)	1/12 (8.3)	1/12 (8.3)	0/12 (0)
21	Hanover (Bornumer Holz)	May 2015	40 (355)	0/40 (0)	24/40 (60.0)	0/40 (0)	0/40 (0)	0/40 (0)
22	Hanover (Mecklenheide)	May 2017	17 (17)	2/17 (11.8)	1/17 (5.9)	1/17 (5.9)	0/17 (0)	1/17 (5.9)
23	Hanover (Mecklenheide)	May 2017	40 (154)	1/40 (2.5)	2/40 (5.0)	0/40 (0)	0/40 (0)	0/40 (0)
24	Hanover (Ricklinger Teiche)	May 2017	40 (805)	2/40 (5.0)	0/40 (0)	0/40 (0)	0/40 (0)	0/40 (0)
25	Hanover (Mecklenheide)	Jun 2017	40 (138)	1/40 (2.5)	1/40 (2.5)	0/40 (0)	0/40 (0)	0/40 (0)
26	Hanover (Georgengarten)	Jun 2017	40 (106)	0/40 (0)	3/40 (7.5)	0/40 (0)	0/40 (0)	0/40 (0)
27	Emen	Sep 2017	40 (55)	2/40 (5.0)	0/40 (0)	0/40 (0)	0/40 (0)	0/40 (0)
28	Lindwedel	Sep 2017	40 (73)	1/40 (2.5)	3/40 (7.5)	0/40 (0)	1/40 (2.5)	0/40 (0)
29	Hanover (Misburger Wald)	May 2018	40 (87)	5/40 (12.5)	0/40 (0)	0/40 (0)	0/40 (0)	0/40 (0)
30	Hanover (Mecklenheide)	May 2018	17 (36)	2/17 (11.8)	0/40 (0)	0/17 (0)	0/17 (0)	0/17 (0)
31	Hanover (Mecklenheide)	May 2018	40 (145)	1/40 (2.5)	1/40 (2.5)	0/40 (0)	0/40 (0)	0/40 (0)
32	Hanover (Misburger Wald)	Jun 2018	40 (44)	33/40 (82.5)	4/40 (10.0)	0/40 (0)	2/40 (5.0)	0/40 (0)
33	Hanover (Misburger Wald)	Jul 2018	40 (194)	2/40 (5.0)	2/40 (5.0)	0/40 (0)	0/40 (0)	0/40 (0)
34	Hanover (Misburger Wald)	Aug 2018	24 (24)	0/24 (0)	2/24 (8.3)	0/24 (0)	0/24 (0)	0/24 (0)
35	Mellendorf	May 2018	40 (94)	2/40 (5.0)	37/40 (92.5)	0/40 (0)	1/40 (2.5)	0/40 (0)
36	Mellendorf	May 2018	40 (341)	3/40 (7.5)	3/40 (7.5)	0/40 (0)	0/40 (0)	0/40 (0)
37	Mellendorf	May 2018	40 (138)	1/40 (2.5)	39/40 (97.5)	1/40 (2.5)	1/40 (2.5)	1/40 (2.5)
38	Mellendorf	May 2018	40 (45)	0/40 (0)	8/40 (20.0)	0/40 (0)	0/40 (0)	0/40 (0)
39	Mellendorf	May 2018	40 (128)	1/40 (2.5)	14/40 (35.0)	0/40 (0)	1/40 (2.5)	0/40 (0)
40	Mellendorf	May 2018	40 (72)	1/40 (2.5)	9/40 (22.5)	0/40 (0)	0/40 (0)	0/40 (0)
41	Mellendorf	May 2018	40 (91)	1/40 (2.5)	2/40 (5.0)	0/40 (0)	0/40 (0)	0/40 (0)
42	Mellendorf	May 2018	40 (102)	13/40 (32.5)	27/40 (67.5)	0/40 (0)	9/40 (22.5)	0/40 (0)
43	Mellendorf	May 2018	40 (83)	11/40 (27.5)	28/40 (70.0)	0/40 (0)	11/40 (27.5)	0/40 (0)
44	Mellendorf	May 2018	40 (87)	2/40 (5.0)	28/40 (70.0)	0/40 (0)	1/40 (2.5)	0/40 (0)
45	Mellendorf	May 2018	40 (68)	2/40 (5.0)	5/40 (12.5)	0/40 (0)	0/40 (0)	0/40 (0)
46	Kassel (Erlenloch)	May 2018	40 (215)	0/40 (0)	1/40 (2.5)	0/40 (0)	0/40 (0)	0/40 (0)
47	Kassel (Niederelsungen)	May 2018	40 (1643)	1/40 (2.5)	2/40 (5.0)	0/40 (0)	0/40 (0)	0/40 (0)
48	Kassel (Niederelsungen)	Jun 2018	40 (45)	0/40 (0)	0/40 (0)	0/40 (0)	0/40 (0)	0/17 (0)
49	Uchte (Rauher Busch)	Jun 2018	40 (65)	0/40 (0)	27/40 (67.5)	0/40 (0)	0/40 (0)	0/40 (0)
50	Wathlingen (Brand)	May 2018	40 (43)	4/40 (10)	3/40 (7.5)	0/40 (0)	1/40 (2.5)	0/40 (0)
Total			1500 (5855)	137/1500 (9.1)	341/1500 (22.7)	3/1500 (0.2)	36/1500 (2.4)	2/1500 (0.1)

^a^Including coinfections

*Abbreviations*: n, number of infected larvae; N, number of examined larvae

### Detection of bacterial pathogen DNA

Regarding pathogen prevalence, 137/1500 larvae (9.1%, 95% CI: 7.7–10.7%) from 39/50 nests (78.0%, 95% CI: 64.0–88.5%) were positive for *Borrelia* spp., while 341/1500 (22.7%, 95% CI: 20.6–24.9%) larvae from 39/50 nests (78.0%, 95% CI: 64.0–88.5%) contained *Rickettsia* spp. DNA. *Anaplasma phagocytophilum* DNA was detected in 3/1500 larvae (0.2%, 95% CI: 0–0.6%) from three different nests (6.0 %, 95% CI: 1.3–16.5%).

**Fig. 2:**
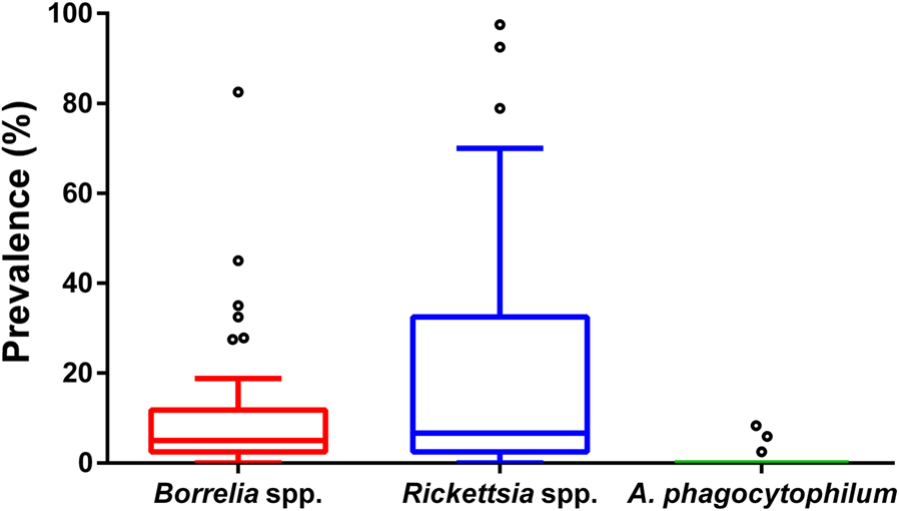
Prevalence of *Borrelia* spp., *Rickettsia* spp. and *A. phagocytophilum* in 50 *I. ricinus* larvae nests. Boxes extend from the 25th to the 75th percentile, with a line at the median and whiskers extending to 1.5 the interquartile range. Circles represent data points outside of this range (outliers)

**Fig. 3:**
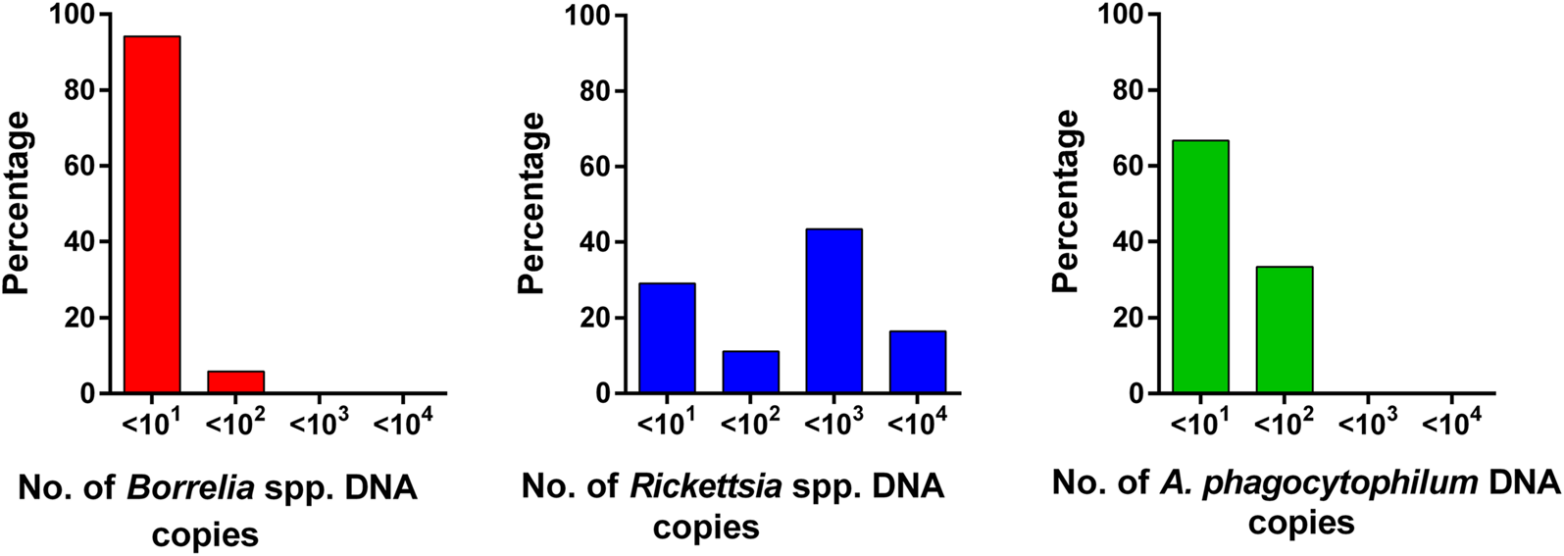
Distribution of *Borrelia* spp., *Rickettsia* spp. and *A. phagocytophilum* gene copy numbers in DNA samples from positive *I. ricinus* larvae

On average, the mean assumed transovarial transmission rate, i.e. the proportion of positive larvae among the total number of examined larvae of the nest, was 12.3% [SD: 15.4%; min. 2.5% (1/40 larvae), max. 82.5% (33/40 larvae)] in *Borrelia*-infected nests, for *Rickettsia*-infected nests 26.5% [SD 29.0%; min. 2.5% (1/40), max. 97.5% (39/40)] and for *A. phagocytophilum*-infected nests 5.6% [SD 2.9%; min. 2.5% (1/40), max. 8.3% (1/12)]. Prevalences of the different pathogens are pictured in Fig. 2, while detailed data per larvae nest are presented in Table 1. Regarding bacterial abundance in *Borrelia*-positive larvae, 94.2% (129/137, 95% CI: 88.8–97.4%) of samples contained ≤ 10^1^*5S-23S* IGS copies and 5.8% (95% CI: 2.6–11.2%) of samples contained between 10^1^ and 10^2^ copies (8/137). On average, 2.7 × 10^0^*5S-23S* IGS copies were detected in positive DNA samples. For *Rickettsia* spp., 29.0% (99/341, 95% CI: 24.2–34.2%) of samples contained ≤ 10^1^*gltA* gene copies, 11.1% (38/341, 95% CI: 8.0–15.0%) of samples contained ≤ 10^2^ copies, 43.4% (148/341, 95% CI: 38.1–48.8%) of samples contained ≤ 10^2^ copies, 16.4% (56/341, 95% CI: 12.7–20.8%) of samples contained between 10^3^ and 10^4^ copies. On average, positive ticks contained 5.4 × 10^2^ copies. The mean copy number of DNA samples of the *A. phagocytophilum*-infected larvae was 2.4 × 10^1^, with 66.7% (2/3, 95% CI: 9.4–99.2%) samples containing ≤ 10^1^*msp2/p44* gene copies and 33.3% (1/3, 95% CI: 0.8–90.6%) between 10^1^ and 10^2^ copies. Copy number distribution for the different pathogens is graphically represented in Fig. 3. Regarding *Borrelia* prevalence in larvae, no significant differences between the different sampling locations were found (Table 2). In contrast, significantly more larvae were infected with *Rickettsia* spp. in Mellendorf 2018 than in Hanover 2010, 2017 and 2018 (Table 3).

**Table 2:** Results of the GLMM testing the influence of location/year and coinfection in the nest on *Borrelia* spp. prevalence in larvae collected in Hanover, Hamburg and Mellendorf (*n* = 1220 larvae from 43 nests)

Factor	Estimate	SE	*z*	*P*
Intercept	− 4.143	0.724	− 5.724	**< 0.001**
Hamburg 2011 *vs* Hanover 2010	0.787	0.742	1.060	0.890
Hanover 2015 *vs* Hamburg 2011	− 0.846	1.203	− 0.703	0.980
Hanover 2017 *vs* Hamburg 2011	− 1.206	0.783	− 1.540	0.622
Hanover 2018 *vs* Hamburg 2011	0.490	0.681	0.719	0.978
Hanover 2015 *vs* Hanover 2010	− 0.059	1.286	− 0.046	1.000
Hanover 2017 *vs* Hanover 2010	− 0.419	0.909	− 0.461	0.997
Hanover 2018 *vs* Hanover 2010	1.277	0.811	1.574	0.599
Hanover 2017 *vs* Hanover 2015	− 0.360	1.313	− 0.274	1.000
Hanover 2018 *vs* Hanover 2015	1.336	1.246	1.072	0.886
Hanover 2018 *vs* Hanover 2017	1.696	0.854	1.986	0.334
Mellendorf 2018 *vs* Hanover 2010	− 0.191	0.756	− 0.253	1.000
Mellendorf 2018 *vs* Hanover 2015	− 0.132	1.211	− 0.109	1.000
Mellendorf 2018 *vs* Hanover 2017	0.228	0.796	0.286	1.000
Mellendorf 2018 *vs* Hanover 2018	− 1.468	0.699	− 2.102	0.272
Mellendorf 2018 *vs* Hamburg 2011	− 0.979	0.559	− 1.750	0.481
Coinfection present in the nest	1.528	0.574	2.662	**0.008**

*Notes*: The full model was significantly different from a null model containing only the random factor “nest ID” (*χ*^2^ = 14.0, *df* = 6, *P* = 0.029). Multiple comparisons between levels of the factor “location/year” were performed using Tukeyʼs contrasts with single-step *P*-value adjustment. Significant *P*-values (*P* < 0.05) are shown in bold

*Abbreviation*: SE, standard error

**Table 3:** Results of the GLMM testing the influence of location/year and coinfection in the nest on *Rickettsia* spp. prevalence in larvae collected in Hanover, Hamburg and Mellendorf (*n* = 1220 larvae from 43 nests)

Factor	Estimate	SE	*z*	*P*
Intercept	− 4.786	1.015	− 4.717	**< 0.001**
Hamburg 2011 *vs* Hanover 2010	1.433	0.997	1.437	0.693
Hanover 2015 *vs* Hamburg 2011	1.971	1.304	1.512	0.644
Hanover 2017 *vs* Hamburg 2011	− 1.373	1.001	− 1.372	0.734
Hanover 2018 *vs* Hamburg 2011	− 1.349	0.961	− 1.403	0.715
Hanover 2015 *vs* Hanover 2010	3.404	1.440	2.365	0.161
Hanover 2017 *vs* Hanover 2010	0.060	1.188	0.051	1.000
Hanover 2018 *vs* Hanover 2010	0.084	1.154	0.073	1.000
Hanover 2017 *vs* Hanover 2015	− 3.344	1.427	− 2.343	0.169
Hanover 2018 *vs* Hanover 2015	− 3.320	1.399	− 2.373	0.158
Hanover 2018 *vs* Hanover 2017	0.024	1.148	0.021	1.000
Mellendorf 2018 *vs* Hanover 2010	2.956	0.990	2.986	**0.032**
Mellendorf 2018 *vs* Hanover 2015	− 0.448	1.288	− 0.348	0.999
Mellendorf 2018 *vs* Hanover 2017	2.896	0.984	2.942	**0.036**
Mellendorf 2018 *vs* Hanover 2018	2.872	0.945	3.038	**0.027**
Mellendorf 2018 *vs* Hamburg 2011	1.523	0.709	2.149	0.251
Coinfection present in the nest	1.772	0.748	2.369	**0.018**

*Notes*: The full model was significantly different from a null model containing only the random factor “nest ID” (*χ*^2^ = 28.9, *df* = 6, *P* < 0.001). Multiple comparisons between levels of the factor “location/year” were performed using Tukeyʼs contrasts with single-step *P*-value adjustment. Significant *P*-values (*P* < 0.05) are shown in bold

*Abbreviation*: SE, standard error

Regarding coinfections, 33/50 (66.0%, 95% CI: 51.2–78.8%) nests were positive for *Borrelia* and *Rickettsia* spp., while three (6.0%, 95% CI: 1.3–16.5%) nests contained larvae positive for all three pathogens. On the basis of larvae, coinfections were found in 8.6% (38/443, 95% CI: 6.1–11.6%) of the pathogen-positive larvae corresponding to 2.5% (38/1500, 95% CI: 1.8–3.5%) of the total investigated larvae. *Rickettsia* spp. and *Borrelia* spp. coinfected larvae were found in 32.0% (16/50, 95% CI: 19.5–46.7%) of nests and in 7.5% (36/478, 95% CI: 5.3–10.3%) of the *Rickettsia* spp. and *Borrelia* spp. positive larvae corresponding to 2.4% of all investigated larvae (36/1500, 95% CI: 1.7–3.3%). Coinfection with *Rickettsia* spp. and *A. phagocytophilum* was noted in two larvae from 2/50 nests (4.0%, 95% CI: 0.5–13.7%), corresponding to 0.6% (2/344, 95% CI: 0.1–2.8%) of the *Rickettsia* spp. and *A. phagocytophilum* positive larvae and 0.1% (2/1500, 95% CI: 0–0.5%) of all investigated larvae. Detailed information on the distribution of coinfections per nest is presented in Table 1. Furthermore, in nests with different pathogens present, larvae had a significantly higher probability of being infected with *Borrelia* spp. or *Rickettsia* spp. than in nests with a single pathogen present (Tables 2, 3).

### *Borrelia* (geno-)species and *Rickettsia* species identification

*Borrelia* (geno-)species determination by RLB revealed presence of *B. spielmanii* in one larva (1.4 × 10^0^*5S-23S* IGS copies). Unfortunately, the *Borrelia* (geno-)species of the remaining 136 *Borrelia*-positive larvae could not be determined by RLB. Due to the high prevalence of *R. helvetica* [5, 6], only a random sample of 63 *Rickettsia*-positive larvae from 19 nests was subjected to pyrosequencing. *R. helvetica* was identified in 73.0% (46/63, 95% CI: 60.3–83.4%) of these larvae, while species discrimination failed for the remaining 17 *Rickettsia*-positive samples. Successfully identified samples had a mean *gltA* copy number of 6.6 × 10^2^, while the 17 unidentified samples contained 3.2 × 10^2^ copies on average. Of the successfully pyrosequenced larvae, 2.2% (1/46, 95% CI: 0.1–11.5%) contained ≤ 10^1^ copies, 2.2% (1/46, 95% CI: 0.1–11.5%) ≤ 10^2^ copies, 71.7% (33/46, 95% CI: 56.5–84.0%) ≤ 10^3^ copies and 23.9% (11/46, 95% CI: 12.6–38.8%) between 10^3^ and 10^4^ copies. Among the samples which were not successfully sequenced, 47.1% (8/17, 95% CI: 23.0–72.2%) contained ≤ 10^1^ copies, 17.6% (3/17, 95% CI: 3.8–43.4%) contained ≤ 10^2^ copies, 17.6% (3/17, 95% CI: 3.8–43.4%) contained ≤ 10^3^ and 17.6% (3/17, 95% CI: 3.8–43.4%) between 10^3^ and 10^4^ copies.

## Discussion

This study aimed to provide an insight into transovarial transmission of *Borrelia* spp., *Rickettsia* spp. and *A. phagocytophilum* in *I. ricinus* under field conditions by extrapolating from DNA detection rates in questing tick larvae and, by extension, to estimate the potential infection risk for humans and animals by tick larvae bites. Since prevalence studies in central Europe on questing nymphal and adult *I. ricinus* report higher numbers of *Rickettsia*- and *Borrelia*- than *Anaplasma*-positive ticks [7, 40, 43], correspondingly higher numbers of *Rickettsia*- and *Borrelia*-positive nests were expected, regardless of the transovarial transmission efficiency. Indeed, both *Rickettsia* spp. and *Borrelia* spp. were detected in 78.0% of nests, while only 6.0% of nests contained *A. phagocytophilum*-positive larvae. The proportion of nests with *Borrelia*- and *Rickettsia*-infected larvae was considerably higher than expected based on the prevalence of these pathogens in questing adult ticks. In studies from Hamburg 2011 and Hanover 2010 and 2015, *Borrelia* spp. DNA was detected in a total of 34.1% (30.0% adults, 34.5% nymphs) [34], 22.7% (33.3% adults, 20.3% nymphs) [33, 39] and 24.1% (35.4% adults, 19.8% nymphs) [40] of ticks. *Rickettsia* spp. DNA was detected in a total of 52.5% (56.0% adults, 52.1% nymphs) [6], 26.2% (30.4% adults, 25.5% nymphs) [5, 44] and 50.8% (54.1% adults, 49.5% nymphs) [7]. The discrepancy between the number of positive nests, and consequently the number of infected female ticks these nests originated from, compared to the prevalence in questing adult ticks may be due to several factors. For example, prevalences measured in questing adult ticks exclude those infections that arise during the blood meal of the adult *I. ricinus* female before oviposition or, in the case of *Rickettsia* spp., during mating [19]. For *A. phagocytophilum*, a prevalence of 86.1% has been found in engorged adult ticks collected from roe deer compared to only 8.9% in questing adult ticks, while *Rickettsia* spp. were detected in 16.6% of engorged adult ticks and 13.9% of questing adult ticks [45]. These infections may be transmitted transovarially, although not as effectively as infections that were acquired earlier [20]. Furthermore, infected larvae might show a higher questing activity than non-infected larvae, e.g. due to higher need of energy as a consequence of infection, or a pathogen-tick-interaction facilitating questing and in turn facilitating pathogen transmission. Thus, infected larvae may be disproportionately represented in the examined larvae collected by the flagging method. Further studies are needed to investigate these explanatory approaches.

The overall infection rate for unfed *Ixodes* spp. ticks with *A. phagocytophilum* in the prevalence studies in Hamburg 2011, Hanover 2010 and 2015 was 3.6% (2.1% adults, 3.8% nymphs), 3.2% (1.9% adults, 3.6% nymphs) and 3.8% (7.2% adults, 2.4% nymphs), respectively [5–7, 44]. In comparison, 6% of the nests in this study were infected. Possibly, the same assumptions as for *Rickettsia* spp. and *Borrelia* spp. are valid to explain this discrepancy.

Within nests, larval pathogen prevalence differed considerably, ranging between 2.5% and 97.5% for *Rickettsia* spp., 2.5% and 82.5% for *Borrelia* spp., as well as 2.5% and 8.3% for *A. phagocytophilum*. Except for *Rickettsia*-infections in Hanover (2010, 2015, 2018) *vs* Mellendorf (2018), no statistically significant regional differences in larval pathogen prevalence were detected. Additionally, to this influence of region, the differences in *Rickettsia* prevalence within nests might be explained by the degree of rickettsial development in the ovarian tissues of the mother tick at the time of oviposition. Burgdorfer and Brinton [20] described that experimentally infected female ticks with generalised massive infections transmitted *Rickettsia* spp. to 100% of their offspring, and this was also observed regarding *R. helvetica* [46]. However, females with a mild rickettsial infection or in the initial phase, had a lower percentage of infected larvae [20].

Overall, *Rickettsia* prevalence in the collected tick larvae was 22.7%. This is comparable to previous studies, as prevalence of *Rickettsia* spp. in field-collected *Ixodes* larvae in Hanover 2005 and 2010 amounted to 27.3% (24/88) and 16.1% (5/31) [4, 5]. In Germany, *R. helvetica* is the most frequent *Rickettsia* spp. in *I. ricinus* [3–7], and was the only *Rickettsia* spp. that could be defined in larvae in the present study. The proportion of *R. helvetica-*infected female ticks producing at least one positive egg or larva was described as 100% [17]. The high *Rickettsia* DNA detection rates in the present study and the identification of *R. helvetica* in the entire subset of sequenced samples provide evidence for a high transovarial transmission rate for *R. helvetica* in *I. ricinus* under field conditions. This is also supported by the high number of *Rickettsia gltA* copies (average 5.4 × 10^2^ copies) found in the positive larva samples, compared to *Borrelia* spp. (average 2.7 × 10^0^ copies) or *A. phagocytophilum* (average 2.4 × 10^1^ copies). Furthermore, the calculated copy numbers may be underestimated, because the tick DNA samples were tested against a plasmid standard. The plasmid standard represents an ideal template, while the tick DNA template is more complex; therefore, the copy numbers of standard *versus* tick DNA are not fully comparable. However, it should be kept in mind that DNA detection does not necessarily imply that viable bacteria were present in the larvae. The pathogens could have died in the egg, during development from egg to larva or during larval hatching. Furthermore, it is also possible that DNA from dead and lysed pathogens in the mother tick may have entered the eggs and was detected.

In contrast to *Rickettsia* spp., a rather inefficient transovarial transmission is assumed for *B. burgdorferi* (*s.l.*). Nevertheless, van Duijvendijk et al. [47] showed that flagged larvae can transmit *B. afzelii* and *B. miyamotoi* to rodents. Thus, larvae also pose a potential infection risk. *Borrelia*-prevalence in individual, field-collected unfed *I. ricinus* larvae varied from 0% to 25.8% in previous studies [33, 47–49]. This is in line with the overall larval *Borrelia* spp. prevalence of 9.1% determined in the present study. Within positive nests, 12.3% of larvae were positive on average, but rates of up to 82.5% were detected. Likewise, Burgdorfer et al. [50] described a transovarial transmission rate of 60.0% and 100.0% in two *I. ricinus* females infected with *Borrelia* spirochetes. However, in another study, only one of sixteen *B. burgdorferi* (*s.l.*)-infected *I. persulcatus* females could transmit the pathogen *via* eggs to the larvae. The infection rate of these larvae was 7.0% [51]. In contrast, an efficient transovarial transmission from female ticks to more than 90.0% of their larvae has been described for the relapsing fever borrelia *B. miyamotoi* [22, 24]. In individual field-collected larvae, *B. miyamotoi* showed a prevalence of 0–4.2% [22, 47, 52]. Among *Borrelia*-positive *I. ricinus* ticks collected in 2015 in Hanover, *B. miyamotoi* showed a prevalence of 18.2% in successfully differentiated *Borrelia*-positive ticks, subdivided into 20.0% in nymphs, 14.0% in females and 18.2% in males [31]. Thus, larvae infected with *B. miyamotoi* were expected. However, species differentiation using RLB was only successful in one case due to the low number of *5S-23S* IGS copies in *Borrelia*-positive larvae. In this larva originating from a nest with a transovarial transmission rate of 82.5%, *B. spielmanii* was identified. For the remaining nests, it was not possible to determine whether the DNA originated from *B. burgdorferi* (*s.l.*) or *B. miyamotoi*, and thus not possible to investigate whether there was a correlation between rather low or high transmission rates and the two *Borrelia* species. Furthermore, the nests may have been infected with several *Borrelia* (geno-)species, including *B. miyamotoi*. Additionally, transovarial transmission rates of *B. burgdorferi* (*s.l.*) might vary between genospecies. Further laboratory studies examining transovarial transmission in ticks infected with known *Borrelia* (geno-)species are needed to achieve a clearer picture and to assess the potential *Borrelia* infection risk posed by larvae.

Due to the low *A. phagocytophilum* prevalence, there were mainly coinfections with *Borrelia* spp. and *Rickettsia* species. In total, 66.0% of all nests and 2.5% of all larvae were coinfected with *Rickettsia* spp. and *Borrelia* spp., while for nymphs and adult ticks, coinfection rates between 7.3% and 22.9% have been described in northern Germany [4, 37, 40]. Furthermore, larvae had a significantly higher probability of being infected with *Borrelia* spp. or *Rickettsia* spp. when the nest was coinfected, leading to the hypothesis that coinfection of the mother tick promotes transovarial transmission efficiency.

Transovarial transmission of *A. phagocytophilum* is described as absent or inefficient in the literature [16, 25]. A study by Baldridge et al. [53] showed transovarial transmission rates of 10.0–40.0% in *Dermacentor albipictus*, while Jahfari et al. [27] and Hagedorn [54] detected *A. phagocytophilum* in 1.3% of field-collected *I. ricinus* larvae. In Hanover, positive *A. phagocytophilum* larvae have not been detected in previous studies [5, 44]. The detection of *A. phagocytophilum* DNA in three unfed larvae in three different nests in the present study shows that transovarial transmission may be possible, but that it is inefficient. Therefore, a potential risk of *A. phagocytophilum* infection by *I. ricinus* larvae appears to be low.

## Conclusions

Detection of pathogen DNA in questing *I. ricinus* larvae provides evidence for transovarial transmission of *Rickettsia* spp. and *Borrelia* spp. under field conditions. In consequence, *I. ricinus* larvae might serve as a source of human or animal infection with these pathogens. However, further studies investigating the percentage of viable transovarially transmitted *Rickettsia* and *Borrelia* species are necessary. As expected, transovarial transmission of *A. phagocytophilum* was rare.

## Data Availability

The data analysed during this study are included in the article.
